# Annotations

**Published:** 1891-12-26

**Authors:** 


### ANNOTATIONS.

Gold too Dear.

Speaking of hypnotism the *Verulam Review* says: "We say meanwhile without hesitating that, if it be correct as it stands, the obtaining from hypnotism an absolute and unfailing cure for every disease with which mankind ever has been or ever will be afflicted, would be too dearly purchased at such a price." With this verdict persons who have a due sense of human responsibility will all be inclined to agree. Hypnotism abolishes in one person judgment and will, and places that person, for a longer or a shorter time, absolutely under the control of another. We have but to imagine the whole world hypnotised, to perceive at once the whole world mad, non-human, without judgment or will. It is best in considering principles of this kind to push them to their ultimate conclusion. The only person who should be subjected to the abolition of will and judgment are mad persons. Hypnotism is simply the carrying a little further of the principles that are ordinarily applied to the treatment of lunatics in asylums. It should never be practised on sane persons. If the whole world could be cured of all its diseases by this immoral method, the game would not be worth the candle; the gold of health would be purchased too dearly at the cost of the loss of judgment and will.

Eucalyptus for Fevers.

Mr. Brendon Curgenven has published a little pamphlet recording his experiences of the treatment of scarlet fever by the administration of eucalyptus oil internally, and by the application of the oil combined with certain other camphoraceous disinfectants to the skin, with the view of preventing the spread of the fever. The value of eucalyptus oil and vapour is well known. In Africa and other tropical parts of the world it is becoming quite common to plant eucalyptus trees in and near the settlements of white persons. The writer has relatives in Africa who ten years ago planted long avenues with eucalyptus trees round about their mission station. The station has, during the last five or six years, enjoyed a high reputation for health, probably in consequence. It is, in fact, a kind of sanatorium for the whites of Eastern Africa. As long ago as 1881 Sir Joseph Lister stated that "the oil of Eucalyptus globulus, if used properly, is a powerful and perfectly reliable antiseptic, and is also quite unirritating and without toxic effects." It is, indeed, a well-established fact that all the essential and balsamic oils of aromatic trees and plants are excellent antiseptics; they readily volatilise, and in doing so liberate ozone. This power of liberating ozone is possessed in a marked degree by the eucalyptus tree, and also by the aromatic oils which are obtained from it. It is the ozone which is thus liberated that renders eucalyptus so valuable in the sick-room. Mr. Curgenven has taken advantage of these known facts to experiment upon his own patients. The story of the first case on which he tried the eucalyptus as a disinfectant is extremely interesting, and brings out practically all the important points required to be emphasised. He says: "I had for some years given up the idea of disinfecting the skin through inability to find a suitable medium for applying the disinfectant, when, in May of last year, I was in a difficulty as to how I should isolate a child suffering from scarlet fever in a family where there were six other children. The child was a year and nine months old, the youngest of the family. The mother would not hear of its being sent to the hospital, and it was impossible to carry out any isolation in the usual way, as she had to attend to all the wants of the other children. I told her that all the children would have the disease if she kept the child in the house, but she said she would not part with her baby, and would risk the others having it. In this emergency I again thought of disinfecting the child, when I remembered having at my house a sample of Tucker's eucalyptus disinfectant, composed, as before stated, of essential oil and camphors, having no fixed oil nor alcohol in it. I determined to try it, and told the mother how I wished it used. She was to rub the child all over with it night and morning, not omitting any portion of the skin, and to sprinkle the bed and the floor of the room with it, so that the air should smell strongly of the vapour. I also gave the child eucalyptus oil in one-drop doses every four hours in an emulsion. When I first saw the child it had the scarlet fever rash over the face, arms, and upper portion of the body. Its throat was so sore that it refused all food, and had not taken anything for two days; it had not slept during two days and nights, being fretful and crying all the time. On my second visit, the following morning, I found the child sitting up in bed eating a slice of bread and butter. The rash had all gone, and the temperature had gone down from 103 deg. to 100·2 deg. The mother told me that after rubbing the child over with the fluid the previous night it went to sleep and slept five hours, awaking apparently well, taking some milk without any difficulty in swallowing. I was quite astonished to see such a change in the child, and told the mother to go on with the disinfectant, rubbing it over night and morning for three days, then each night, after, a warm bath for four days more. The child was so saturated with the eucalyptus inunction, by inhalation of its volatile vapour, and by medication, that the disease appeared to have been stayed and every germ destroyed as desquamation occurred only on the parts of the skin where the rash was seen. The other children had free access to the room, and none of them took the disease." It is not necessary to say more at the present time than to suggest that all medical men having cases of scarlet fever and other zymotic diseases to treat will consider it an obligation at least to try the method advocated by Mr. Curgenven. If the treatment by encalyptus should prove to be as generally successful as he anticipates, a very important addition will have been made to our medical resources.

Miss F. P. Cobbe on Health and Holiness.

Miss Frances Power Cobbe struggles heroically with what she considers to be some of the greatest evils of her time. A few weeks ago Miss Cobbe read an address to the Cambridge Ladies' Discussion Society on the subject "Health and Holiness." That address has since been published, and we are favoured with a copy. The world has known Miss Cobbe long enough to take the measure of her character and teaching capacity. She is a good woman and a true teacher. Her views on one important subject, that of vivisection, are entirely opposed to those of some men whose characters will bear comparison with Miss Cobbe's, and whose mental capacity is at least as great. Still, it is to be borne in mind that history furnishes us with many examples of eminent and distinguished persons whose work has been condemned as foolish and useless by succeeding generations. It is not very wise to stand by mere names. or to count heads. The thing to do is to take up the highest possible moral and intellectual standpoint, and from that vantage ground to consider and weigh facts and arguments. Miss Cobbe affirms that this is the age of scientific Hygeiolatry; in other words men to-day sacrifice holiness or high character to health. "Great is Hygeia of the sanatarians! There is no goddess but Health. And Dr. Richardson is her prophet." Against this worship of health Miss Cobbe enters her most emphatic protest. She says the position of the "sanitarians," by which she probably means the vivisectionists, may be thus defined. "Every practice which in the opinion of experts conduces to bodily health, or tends to the cure of disease, or to the discovery of the means of curing disease, is *ipso facto* morally lawful and right. This doctrine," says Miss Cobbe, "is ethically false." Whilst she puts a very high value upon health, she still affirms that duty is first in order, and health but second. The question is important from a practical point of view. Stupid people do not see that it is so. But it is. The man who believes in moral obligations at all, will see at once that not only should duty take precedence of health, but that it should take precedence of every other circumstance whatsoever. This is quite fundamental. Of course, when we have settled that point we have not settled anything at all about vivisection. It is not at all unnatural, but very natural, to suppose that many a physiologist vivisects animals because he holds it to be his duty. We often regret that in these days we cannot take a more decided stand on one side or other of the vivisection question. But at least it will be allowed that a judge who has a difficult and complicated case before him is both wise and right in giving as much time as possible to its consideration. We have no hesitation whatever in saying that Miss Cobbe's address is excellent in tone and temper, and also in intelligence and sentiment. All classes of medical men, vivisectionists as well as others, will do well to look at the subject of vivisection from Miss Cobbe's standpoint. It is a painful and a difficult subject, and as no man, and no class of man, can lay any claim to infallibility these are the best of reasons for considering the whole question in the light of every variety of intelligence.

